# A rare case of extramedullary plasmacytoma of the paranasal sinuses

**DOI:** 10.1093/bjrcr/uaag009

**Published:** 2026-04-08

**Authors:** Laiba Butt, Laura Munro, Andrzej Wieczorek, Robert Wotherspoon, Brook Adams

**Affiliations:** York and Scarborough Teaching Hospitals NHS Foundation Trust, The York Hospital, York, YO31 8HE, United Kingdom; York and Scarborough Teaching Hospitals NHS Foundation Trust, The York Hospital, York, YO31 8HE, United Kingdom; Queens Centre for Oncology & Haematology, Castle Hill Hospital, Hull University Teaching Hospitals NHS Trust, Cottingham, HU16 5JQ; York and Scarborough Teaching Hospitals NHS Foundation Trust, The York Hospital, York, YO31 8HE, United Kingdom; York and Scarborough Teaching Hospitals NHS Foundation Trust, The York Hospital, York, YO31 8HE, United Kingdom

**Keywords:** extramedullary plasmacytoma, paranasal sinuses, radiotherapy, myeloma, diffusion weighted imaging, PET

## Abstract

Plasmacytoma is a rare malignant tumor originating from plasma cells either located in the bone marrow (known as solitary bone plasmacytoma or SBP) or from outside the bone, usually arising from the mucosa (known as extramedullary plasmacytoma). Extramedullary plasmacytoma (EMP) in the head and neck region is extremely uncommon, and therefore this case involving the paranasal sinuses is reported for its rarity and to demonstrate the importance of imaging in the diagnosis and follow-up of the disease. Both pathologies can progress to multiple myeloma, a much more common manifestation of the continuum of plasma cell neoplasms.

## Clinical presentation

A 79-year-old male presented with a 1-year history of a painless, 2 cm swelling at the distal aspect of his last standing upper right molar tooth. The swelling had worsened in recent months, prompting a referral by his general dental practitioner via the 2-week wait pathway for suspected head and neck cancer. Additionally, he reported a blocked sensation in his right nostril and occasional nosebleeds.

## Investigations/imaging findings

**Figure 1:**
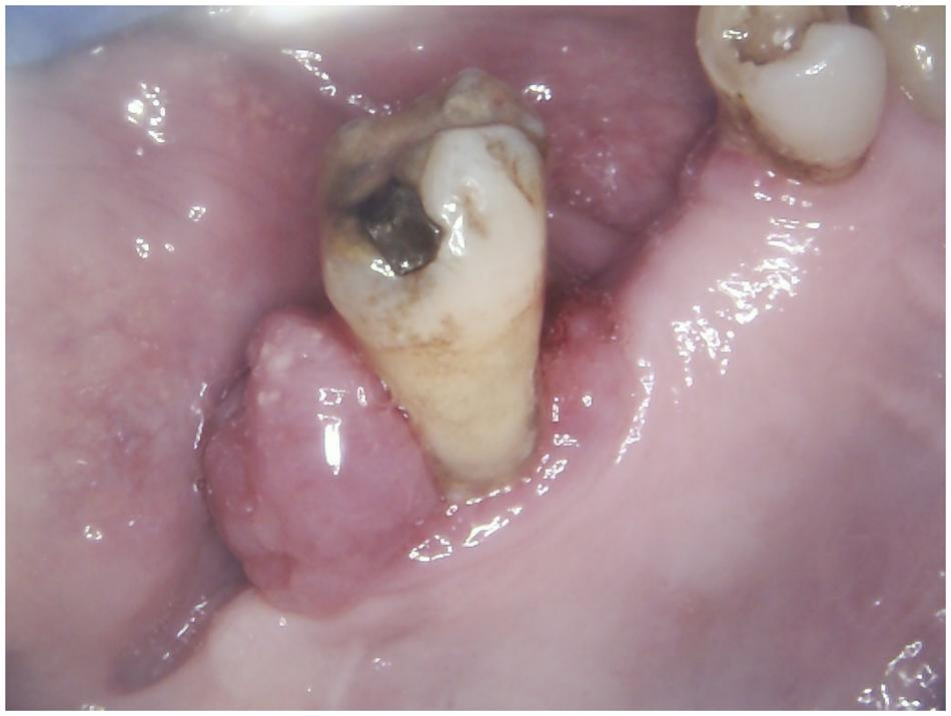
An intraoral photograph demonstrates a focal gingival swelling at the distal aspect of the patient’s upper right 7.

On examination, a 2 cm inflammatory epulis was noted distal to the upper right 7 (UR7), which was grade 3 mobile (figure 1). There was no clinical evidence of cervical lymphadenopathy or hepatosplenomegaly.

**Figure 2:**
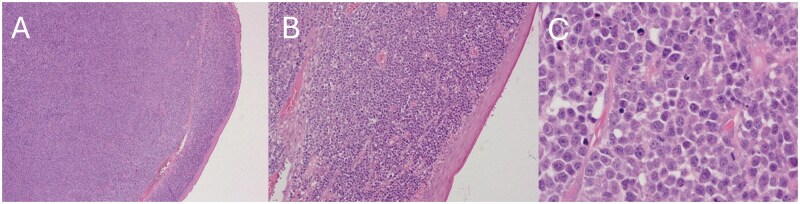
(A-C) Three hematoxylin and eosin images of increasing magnification demonstrating a stroma replaced by a population of atypical lymphoid cells.

The UR7 and associated inflammatory tissue were surgically removed under local anesthetic and sent for histopathology. The excision biopsy showed a dense infiltration of atypical plasma cells with kappa light chain expression, confirming a plasma cell neoplasm. Immunohistochemistry markers were CD138+/−, CD19−, CD319+, IRF4+, kappa-positive plasma cells, and lambda-negative plasma cells, indicating a neoplastic plasma cell phenotype (figure 2). Given the anatomical location, this was reported as an extraosseous plasmacytoma.

Initial blood tests revealed normal immunoglobulins with no paraproteinaemia. Serum-free light chains showed free kappa light chains of 67.76 mg/L (3.30-19.40), free lambda light chains of 20.25 mg/L (5.71-26.30), and a kappa: lambda ratio of 3.346 (0.26-1.65). The slight excess of kappa free light chains was likely originating from the plasmacytoma. Following radiotherapy, the patient’s kappa: lambda ratio normalized.

The eGFR was 59 mL/min/1.72 m^2^. The serum calcium was 2.4 mmol/L (2.20-2.60).

A bone marrow aspirate was multiparticulate and mildly hypercellular. There was evidence of trilineage hematopoiesis without overt dysplasia. Occasional plasma cells and mast cells were seen. The trephine was a very good length with largely preserved normal architecture with sequentially maturing trilineage hematopoiesis with no overt abnormal infiltration.

**Figure 3:**
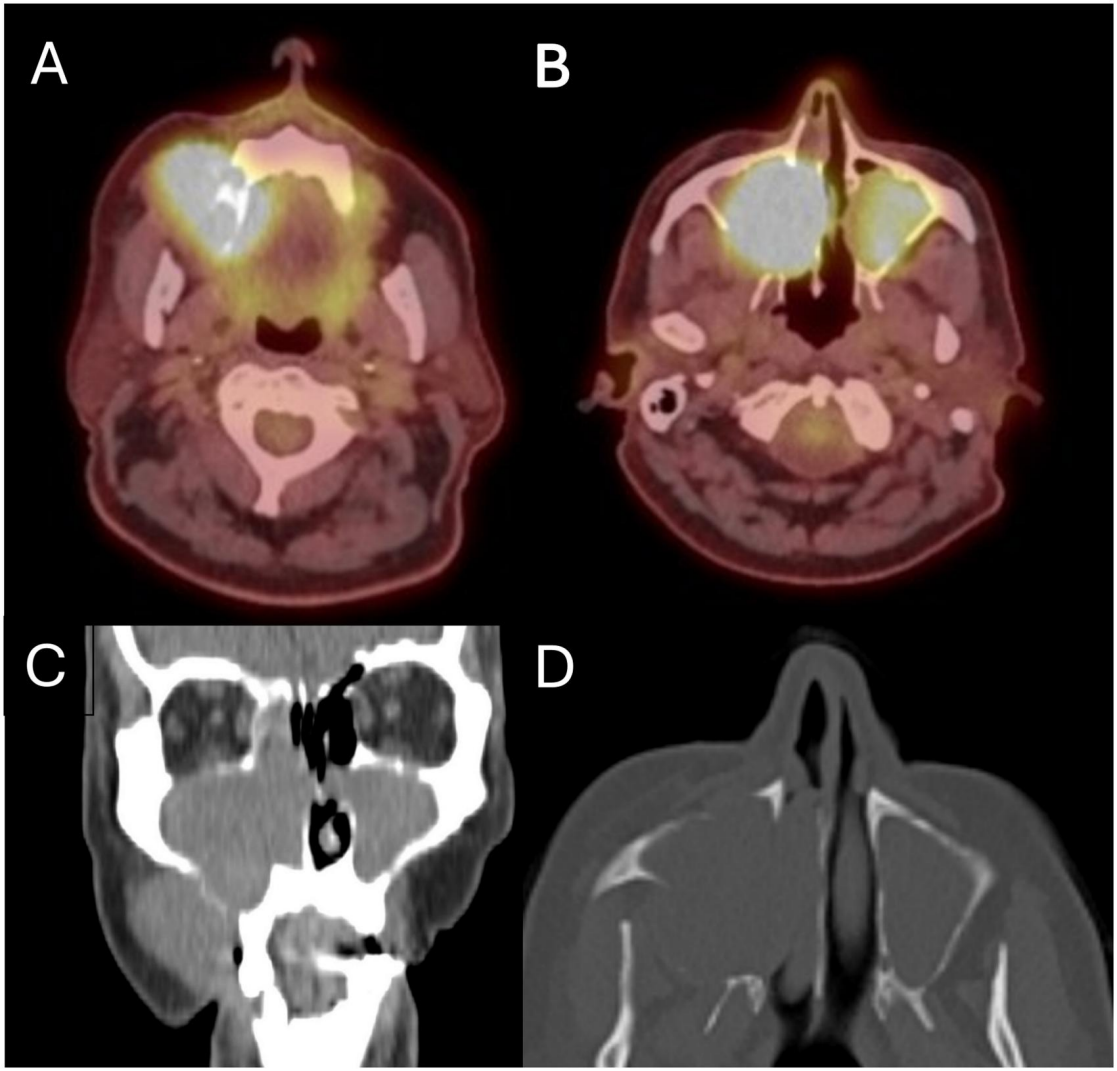
(A and B) A PET-CT study displaying hypermetabolic lesions in both maxillary antra. The degree of FDG-avidity is greater in the right maxillary antrum. (C and D) The non-contrast component of the study shows disease extending through the floor and posterolateral wall of the right maxillary sinus into the buccal sulcus and buccal space respectively.

A total body 18-fluorodeoxyglucose PET-CT study was performed, and this showed an aggressive, intensely hypermetabolic soft tissue lesion in the right maxillary sinus that was eroding through the walls of the sinus. The right maxillary soft tissue had an SUVmax of 10.8 (figure 3). Fluorodeoxyglucose (FDG)-avidity was demonstrated in the soft tissues in the left maxillary antrum and elsewhere within the paranasal sinuses, but the SUVmax was 5.9 and therefore, given the lack of bone erosion it was felt that these appearances likely reflected benign mucosal disease. No FDG-avid lesions were seen in the liver, spleen, or adrenals. No FDG avid retroperitoneal lymphadenopathy was noted. The myocardium, liver, kidneys, renal pelvis, ureters, and bowel showed a physiologic distribution of uptake. No evidence of hypermetabolic bone lesions was found.

**Figure 4:**
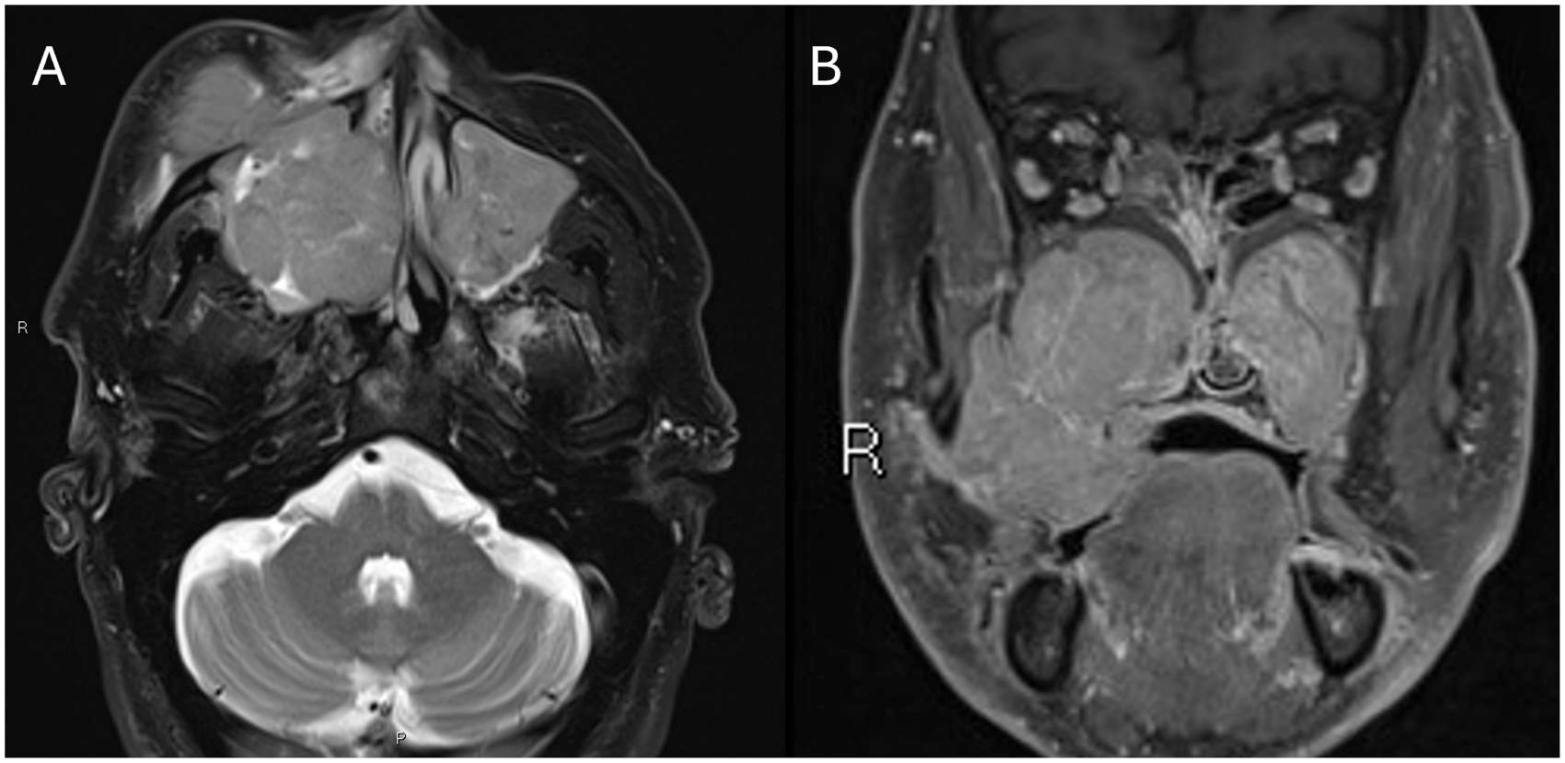
(A) an axial T2 sequence with DIXON fat saturation through the maxillary antra demonstrates bilateral T2 intermediate signal masses. The right sided mass extends beyond the confines of the antrum and the left sided mass does not. (B) a post-contrast fat saturated coronal T1 sequence shows homogenous moderate enhancement with no internal necrosis evident.

An MRI was performed subsequently to better assess the soft tissue extent of the lesion, and this revealed abnormal enhancing soft tissue with a similar intermediate T2 signal within both maxillary sinuses, more extensive on the right side with extension into the nasal cavity and ethmoid labyrinth (figure 4). There was no evidence of intraorbital or intracranial extension nor signs of perineural spread. The largest lesion, associated with the right maxillary sinus, measured maximally 7.4 cm.

## Treatment

The patient received radical radiotherapy with 50 Gy in 25 fractions. The target volume included the tumor with a 6 mm margin, bilateral maxillary sinuses, nasal cavity, ethmoid sinuses, and the right level Ib and II lymph nodal stations.

The patient completed radiotherapy in January 2022 and was subsequently discharged to hematology for follow-up.

## Outcome and follow-up

**Figure 5:**
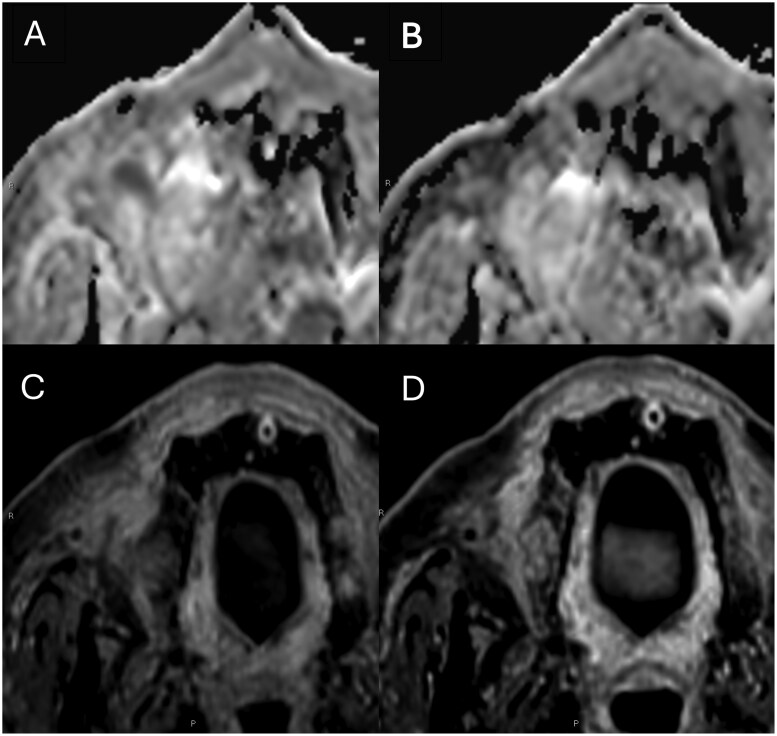
Axial apparent diffusion coefficient and post-contrast axial T1 with fat saturation from March 2022 (A and C) and from July 2022 (B and D) show the utility of diffusion weighted imaging in demonstrating a small restricting/cellular residuum in the right buccal sulcus. The post-contrast imaging in isolation does not enable the distinction between disease residuum and radiotherapy-related/mucosal enhancement to be made.

An MRI performed post-radiotherapy in March 2022 revealed a very good response to treatment, with almost complete resolution of the left maxillary antral mass. A small residual lesion was noted in the right buccal sulcus, measuring approximately 19 × 16 mm, with moderate diffusion restriction suggesting a small cellular residuum (figure 5). An additional right maxillary antral mucosal retention cyst and benign-appearing nasal polyps were observed.

A subsequent MRI in July 2022 showed no evidence of residual or recurrent disease. There was expected mucosal thickening in both maxillary antra, and the previously noted diffusion restriction in the right buccal sulcus was no longer visible, indicating a complete cellular response.

Rates of progression to myeloma in patients with EMP vary from 11% to 30% at 10 years. The overall survival rate at 10 years is 70%.^1^ This is in distinction to SBP, where the prognosis is poorer with a rate of progression of 50%-60% at 10 years and a median overall survival time of 10 years.^2^

The patient is currently having 6 monthly clinical follow-ups with serum electrophoresis and free light chains, which have remained within normal limits to date. He remains in remission for 3 years post-radiotherapy. Further imaging is only to be performed if there is biochemical or symptomatic relapse.

## Discussion

This case highlights the presentation and management of an EMP in the head and neck region.

EMPs are a rare pathology in the head and neck, accounting for <1% of head and neck malignancies.^3^ The incidence is higher in men with a mean age of 60-80 years. There is a male-female predilection of 3:1 and no clear racial predilection. EMPs are mainly found in the sinonasal or nasopharyngeal regions (75%), followed by the oropharynx and larynx, but have also been reported in even rarer sites such as the minor salivary glands, hypopharynx, thyroid, parotid glands, and even the middle ear.^4–6^

EMPs usually present as a slow-growing mass, and presentation is therefore very variable depending on location. Lesions of the sinonasal region can reach a significant size without noticeable symptoms, leading to delayed diagnosis. In our case, only the oral cavity component of the lesion was detected clinically, which was the tip of the iceberg. The most common clinical features for sinonasal lesions are soft tissue swelling and nasal obstruction (80%).^7^ Other potential presenting symptoms include epistaxis, nasal discharge, pain, proptosis, cervical lymphadenopathy, and cranial nerve dysfunction.

Securing the diagnosis biochemically is challenging, as only 25% of patients have a raised M-protein in their plasma or urine, but if raised at presentation, serial measurements can be useful as part of the follow-up strategy. Macroscopically, EMPs have variable appearances. They can appear as a yellowish-gray to dark red and can be sessile, polypoid, or pedunculated lesions.

Our case demonstrates that EMPs, especially in the maxillary sinuses, require careful clinical and radiographic evaluation to confirm the diagnosis and to rule out other potential malignancies. MRI is best placed to delineate the extent of the lesion in the paranasal sinus and helps distinguish malignant disease from benign mucosal disease, which can be challenging to determine on both CT and PET-CT. Beyond this, appearances are generally nonspecific with the differential including squamous cell carcinoma, lymphoma, minor salivary gland tumors, and granulomatosis with polyangiitis. Biopsy provides this distinction. MRI with diffusion-weighted imaging can be useful for assessing treatment response, with DWI demonstrating small-volume cellular residuum in this case.

Once the diagnosis has been established, if the EMP is readily accessible, surgical resection can be considered, but, in the head and neck region, this is usually not the treatment of choice due to difficult accessibility, such as lesions in the sinuses and temporal bones.^8^ EMPs have been shown to be highly radiosensitive, and radiotherapy obviates the need for potentially mutilating surgery.^9^^,^^10^ The United Kingdom Myeloma forum recommendations suggest a radiotherapy dose of 40 Gy in 20 fractions for tumors <5 cm and up to 50 Gy in 25 fractions for tumors ≥5 cm. If cervical nodes are involved (or in Waldeyer’s ring tumors), these should be included in the radiotherapy field.^11^

This case underscores the importance of timely histopathological assessment, PET-CT and MRI, and a tailored radiotherapy approach in managing EMPs.

## Learning points

- Extramedullary plasmacytoma is a rare variant of plasma cell neoplasm that can occur in the head and neck/paranasal sinuses.
- PET-CT is a key part of excluding multiple myeloma but struggles to differentiate malignant disease from inflammatory mucosal disease in the paranasal sinuses.
- MRI is useful for delineating disease extent, and when diffusion-weighted imaging is used as part of the follow-up protocol, it has a high sensitivity and specificity for defining small volume cellular residuum.
- Radiotherapy is the preferred treatment modality for extramedullary plasmacytoma of the head and neck due to poor tumor surgical accessibility and the proven radiosensitivity of the disease.
